# A Chamber-Based Digital PCR Based on a Microfluidic Chip for the Absolute Quantification and Analysis of KRAS Mutation

**DOI:** 10.3390/bios13080778

**Published:** 2023-08-01

**Authors:** Jie Ren, Gangwei Xu, Hongna Liu, Nongyue He, Zhehao Zhao, Meiling Wang, Peipei Gu, Zhu Chen, Yan Deng, Dongping Wu, Song Li

**Affiliations:** 1 Hunan Key Laboratory of Biomedical Nanomaterials and Devices, Hunan University of Technology, Zhuzhou 412007, China; m20077700011@stu.hut.edu.cn (J.R.);; 2 State Key Laboratory of ASIC and System, School of Microelectronics, Fudan University, Shanghai 200433, China; 3 Hunan Shengzhou Biotechnology Company Limited, Shanghai 200439, China; 4 Hengyang Medical School, University of South China, Hengyang 421001, China

**Keywords:** microfluidic chip, KRAS gene, glass-PDMS-glass sandwich, chamber-based digital PCR

## Abstract

The Kirsten rat sarcoma virus gene (KRAS) is the most common tumor in human cancer, and KRAS plays an important role in the growth of tumor cells. Normal KRAS inhibits tumor cell growth. When mutated, it will continuously stimulate cell growth, resulting in tumor development. There are currently few drugs that target the KRAS gene. Here, we developed a microfluidic chip. The chip design uses parallel fluid channels combined with cylindrical chamber arrays to generate 20,000 cylindrical microchambers. The microfluidic chip designed by us can be used for the microsegmentation of KRAS gene samples. The thermal cycling required for the PCR stage is performed on a flat-panel instrument and detected using a four-color fluorescence system. “Glass-PDMS-glass” sandwich structure effectively reduces reagent volatilization; in addition, a valve is installed at the sample inlet and outlet on the upper layer of the chip to facilitate automatic control. The liquid separation performance of the chip was verified by an automated platform. Finally, using the constructed KRAS gene mutation detection system, it is verified that the chip has good application potential for digital polymerase chain reaction (dPCR). The experimental results show that the chip has a stable performance and can achieve a dynamic detection range of four orders of magnitude and a gene mutation detection of 0.2%. In addition, the four-color fluorescence detection system developed based on the chip can distinguish three different KRAS gene mutation types simultaneously on a single chip.

## 1. Introduction

Single nucleotide polymorphism (SNP) is extremely important for tumor diagnosis and typing [1,2,3,4]. At present, the technologies to detect SNPs mainly include next-generation sequencing (NGS) technology and polymerase chain reaction (PCR) technology. Due to its high throughput, NGS technology can achieve multi-target detection and has been widely used in clinical practice [5,6]. However, with the high cost of NGS, complicated operation processes, and long turnaround time, its application is limited in clinical detection. PCR technologies mainly include real-time quantitative PCR (qPCR) and dPCR. qPCR has been widely used in the clinical field and plays an important role in gene mutation detection; furthermore, qPCR-derived allele-specific PCR also has been widely used for SNP detection [7,8,9,10,11]. However, the detection of qPCR is limited when detecting low-abundance mutation samples; due to the interference of wild genes, detection can be missed [9,12]. dPCR is an absolute nucleic acid quantification technology. Due to its “divide and conquer” technical principle, it has higher quantitative precision and sensitivity than qPCR and is more suitable for the detection of SNPs.

The KRAS gene is the most common proto-oncogene in humans; it instructs cells to grow, mature, divide, and specialize in functions. KRAS gene mutations occur in 90% of pancreatic cancers, 30–50% of colon cancers, and 15–20% of lung cancers (mostly NSCLC) [13]. Meanwhile, KRAS gene mutations also appear in cancer types such as cholangiocarcinoma, cervical cancer, bladder cancer, liver cancer, and breast cancer. The mutation frequency of the KRAS gene in different cancers is shown in Table 1. Most KRAS proto-oncogene mutations occur on the 12th and 13th codons [13,14,15]. KRAS gene mutations are tolerant to targeted drugs against EGFR mutations and are an extremely defective type of drug-resistant mutation [16,17,18]. There are six types of mutation at the 12th codon, accounting for about 13–14% of the total types of mutation. The detection and typing of KRAS gene mutation not only has important clinical significance for the treatment and prevention of cancers, but also has essential value for guiding the development of cancer drugs.

When dPCR was first proposed in 1999, it had been used to detect mutations in the KRAS gene. dPCR places a single DNA molecule in a separate reaction chamber and amplifies it via PCR to detect a specific target sequence, using either the TaqMan probe method or the dye method. Therefore, chamber-based digital PCR detection mainly includes three steps: first, the PCR reaction mixture is dispersed to tens of thousands of reaction units (reaction chambers) through a specific technology; second, single molecules are amplified in the reaction chamber; third, the absolute quantification of nucleic acid molecules is achieved by detecting the fluorescence signal after PCR and, finally, calculating the copy number of the sample by Poisson distribution statistics. However, due to the low sample throughput and high contamination risk, early dPCR techniques were not successfully used in the clinical domain. In recent years, dPCR has been further developed with the help of microfluidic chips based on micro electro mechanical system (MEMS) technology. The dPCR platforms are mainly classified into two groups: water-in-oil dPCR and microchamber array dPCR. These platforms greatly simplify the dPCR process, allowing better feasibility. However, most commercial platforms currently use droplet digital PCR (ddPCR). dPCR can perform absolute quantization without the need for standard curves, but the detection range is narrow, the targets that can be detected in a single pass are limited, the operation is complex, and the equipment and reagents are expensive. With the exception of multi-fluorescence channel dPCR platforms developed by some biotechnology companies (such as Stilla technologies and Biorad), most commercial platforms generally have only two fluorescence channels, which can only detect a limited number of targets at a time. The ability to perform multiple actions can only be demonstrated by using different fluorescence probe concentrations and mixing different fluorescence probes with the same target. For multi-mutant KRAS genes, multiple detections are required to meet the needs of genotyping [19,20]. Tanaka et al. developed a multiplex dPCR detection technology consisting of a dual-colored real-time fluorescence platform that allows detection of multiple mutation types at once [15]; however, the technology suffers from the disadvantage of low detection precision (selectivity of about 1%). In addition, freely dispersed droplets in droplet digital PCR can also fuse together due to thermal motion during amplification, which affects the monodisperse nature of the droplets and, thus, the accuracy of the quantization.

In contrast to droplet dPCR platforms, chamber-based dPCR platforms achieve reagent partitioning by dispensing samples into a large number of tiny reaction chambers with equal volume, generating droplets that are both uniform and stable. Due to interfacial wetting and thermal oscillation, the droplets are isolated from one another [12,19,20,21,22]. In addition, due to low functional integration, most custom and commercial dPCR platforms typically consist of multiple device units, including a droplet generator and one reader, making the overall system large, complex, and expensive [22]. Moreover, the maintenance costs and consumable items of these commercial dPCR devices may exceed the affordability of general laboratories in resource-limited areas. Therefore, a cost-efficient and portable dPCR device with high-function integration is urgently demanded for nucleic acids analysis in resource-limited settings [23]. In addition, microchamber-based on-chip dPCR can be directly imaged by a relatively simple fluorescence microscope, which is convenient for data quality control. The chip products can be stored in an undisturbed storage environment for more than 12 months, and the stored chip products are still usable. It has advantages of flexibility, portability, and low cost, along with high accuracy and repeatability [13,24,25]. Because of its various advantages, such as small sample volume and fast reaction speed, microfluidic technology has become an ideal platform for the development of low-cost and high-accuracy disease diagnosis technology [26,27]. Microfluidic chips have been widely developed and applied in recent years [28].

The use of multicolor dPCR detection technology spans across a variety of applications, including detection of SNPs [29,30], clinical diagnostics, oncology [31,32], environmental monitoring [33,34], single-cell analysis [35,36] and food or agricultural testing [37,38]. dPCR also serves as a supplement to qPCR [39,40], NGS [41], prenatal testing [42,43], and copy number variation (CNV) genotyping [44,45]. Currently, dPCR technologies that have been commercialized are capable of meeting applications in a variety of settings; however, a number of critical challenges remain, such as the dPCR system has high cost and complex operation and the cost of chip processing is high. Nevertheless, dPCR is currently a mature technology and is expected to be further driven by an increase in targeted and genetic diseases (e.g., infectious diseases, cancer, HIV, etc.). dPCR allows for a more cost-effective and simpler way for physicians and researchers to detect and analyze the results of tumor samples, as it provides an absolute quantification and reduces inter-laboratory variability and errors [46].

In this work, we developed a microfluidic chamber array chip, which uses PDMS flexible material as the structural layer, glass as the structural reinforcement layer, and a chamber-sealing layer. It utilizes a flexible valve to ensure that the chip can automate the segmentation and division of samples. In addition, a high-efficiency KRAS gene mutation detection system was developed based on a four-color fluorescence system; it can simultaneously detect and quantify three KRAS gene mutations on one chip. The absolute quantification of nucleic acid and detection of the KRAS mutant gene was verified on the developed detection system.

## 2. Materials and Methods

### 2.1. Microfluidic Chip Design and Fabrication

**Figure 1:**
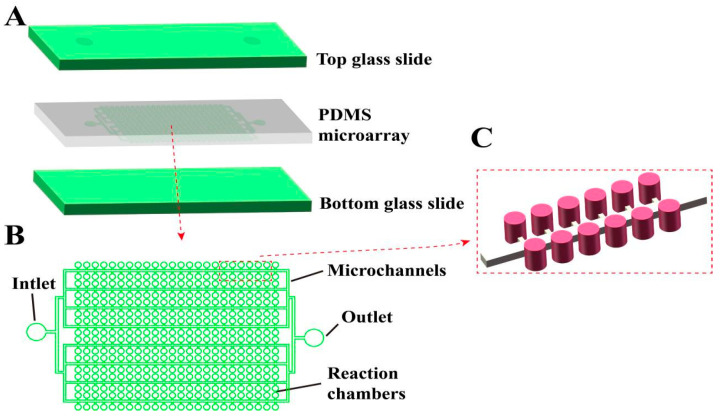
Schematic diagram of the structure of our integrated microchamber array dPCR chip. (**A**) Schematic diagram of the three-layer, glass–PDMS–glass structure of the chip. (**B**) Top view of the internal microstructure of the chip. (**C**) 3D schematic diagram of the internal microstructure of the chip.

As shown in Figure 1A, our newly designed chip consists of a three-layer structure, with a glass substrate on both the top and bottom, and a PDMS microarray structure layer in the middle [47,48]. The sandwich structure of the glass–PDMS–glass design not only ensures the stability of the chip structure, but also effectively prevents the evaporation of the liquid during the thermal cycle [49]. The glass allows light transmission, which assists in reading the fluorescent signal inside the chamber after amplification. As shown in Figure 1B,C, the internal structure of the chip consists of a sample inlet, a sample outlet, a microchamber array, and a microchannel. All the chamber arrays inside the chip are evenly distributed on both sides of the microchannel, with one microchannel realizing the liquid introduction of two columns of chamber arrays at the same time. Microchambers are used to store liquid reagents and serve as separate reaction compartments. The microchannel ensures that the chip sample can smoothly enter the chamber and is subsequently filled with an oil phase to serve the purpose of separating the chamber. Our chip has 54 parallel microchannels and 108 rows of chamber arrays, each with 198 microchambers. The total number of chambers contained in each chip is 21,384, similar to the number of partitions in existing commercial dPCR platforms, and can meet the requirements of most dPCR quantitative experiments and rare mutation detection applications. The main channel width is 60 μm, and its height is 55 μm. The diameter of the microchamber is 87 μm, the height is 120 μm, and the single-chamber volume is about 0.71 nL, and the chip thus allows more than 30 µL of reagent to be delivered. This design not only ensures the fluency of sample injection, but also reduces dead volume and waste of samples. The sample inlet and outlet are symmetrically designed, which is convenient for experimental operation and commercial application. As shown in Table S2, the main materials and instruments used in the chip preparation process are listed.

**Figure 2:**
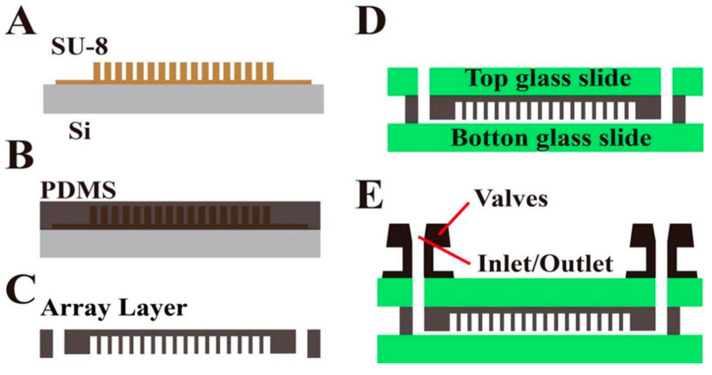
Process flow chart of chip preparation. (**A**) Schematic diagram of SU-8 mold; the prepared silicon chip mold is inverted to prepare the flexible PDMS chip structure layer. (**B**) Pouring PDMS over the master mold and heat curing. (**C**) PDMS structural layer peeled and punched, releasing the PDMS replica. (**D**) PDMS structure layer is bonded with a clean glass cover and substrate by oxygen plasma bonding to prepare the microchamber array chip with “sandwich” structure. (**E**) In order to facilitate automatic liquid separation, a flexible valve is fitted at the chip inlet/outlet.

The chip preparation process is shown in Figure 2. First, the prepared silicon chip mold is inverted to prepare the flexible PDMS chip structure layer; then, the PDMS structure layer is bonded with a clean glass cover and substrate by oxygen plasma bonding to prepare the microchamber array chip with “sandwich” structure. Finally, in order to facilitate automatic liquid separation, a flexible valve is fitted at the chip inlet/outlet.

### 2.2. Preparation of Chip Mold

First, use design software AutoCAD (https://www.autodesk.com, accessed on 14 June 2022) to design the chip mask. Since the chip’s fluid channel and the chip’s chamber have different heights, the chip mold is prepared by a double-layer photolithography process, while the channel layer and the chamber structure layer need to be designed on two-layer masks. The well-designed masking pattern is printed on the glass as a photolithographic mask using a high-resolution printer. The chip mold adopts a 4-inch monocrystalline silicon wafer as substrate, and adopts SU8 photoresist (MicroChem, Adel, GA, USA) for the structural layer. SU8 photoresist has excellent photolithographic processing properties, is suitable for the preparation of microstructures with high aspect ratios, and is very suitable for the preparation of microfluidic chip molds [50,51]. The mold preparation process of the chip is the standard soft photolithography technique. First, the microchannel structure layer is fabricated on the silicon wafer. After the microchannel structure layer is fabricated, the microcavity layer is prepared.

The SU-8 chip mold is successfully fabricated by photolithography using the designed mask. The chip structure size is measured by a three-dimensional optical system. The chip fluid channel layer size is 59.2 ± 0.4 μm, and the chip chamber layer thickness is 120.3 ± 2.7 μm (the average height of 100 chambers is randomly measured). Under the optimized glue leveling parameters and lithography process conditions, the structure size of the prepared chip is the same as the designed size. Subsequently, the microfluidic chips required for the experiment are successfully prepared by using the chip mold according to the chip preparation process described by Section 2.1. The whole chip has a “sandwich” structure, high structural stability, and low preparation cost.

### 2.3. Chip Operation

**Figure 3:**
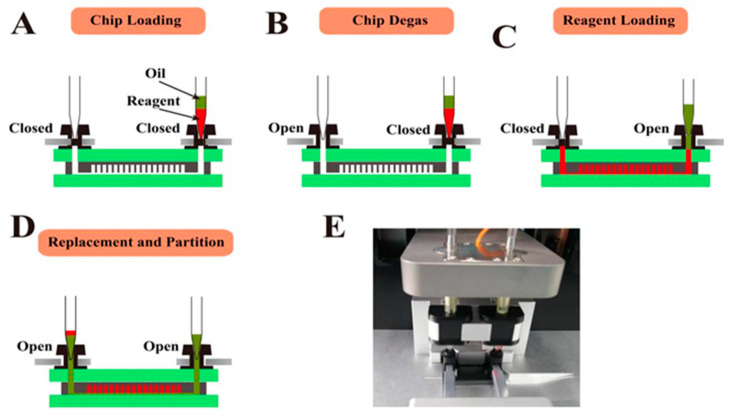
Cross-section view of chip operation process and physical diagram of device. (**A**) Loading the chip in the Chip Loader device. (**B**–**D**) Degassing the chip in a vacuum pump attached to its outlets and then aspiration of the sample into the microchannels under the negative pressure and the oil into the microchannels to replace and partition the samples. (**E**) Photograph of the chip loader device.

**Figure 4:**
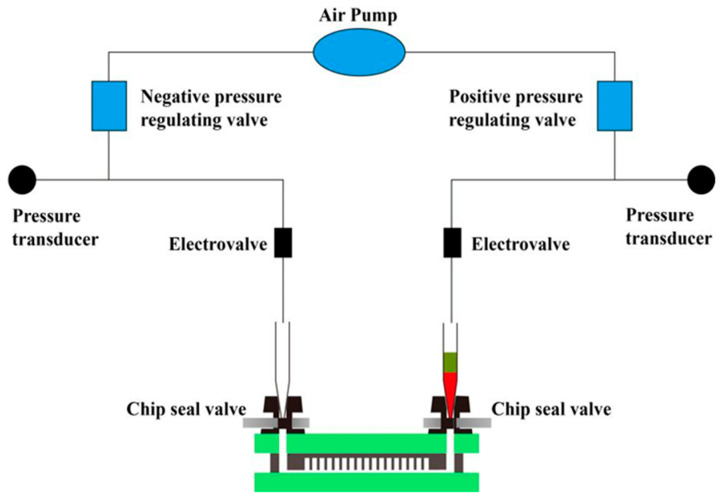
Schematic diagram of automatic chip droplet preparation system. The actuator is controlled by the main control board and the upper computer software. This system is divided into four steps: Chip loading, Chip degassing, Incoming liquid phase, Inlet oil phase (reagent split). The gas path system of the automatic chip sampler can complete the process of automatic sampling and droplet segmentation.

The microfluidic chip designed in this section can realize automatic reagent segmentation, allowing the automatic injection and droplet segmentation process to be completed on the automatic chip sampler (Shengji Gene Technology Co., Ltd., Xuzhou, China. SXXB No. 20190036), as shown in Figure 3. The basic principle of the automated chip sampler’s gas path system is shown in Figure 4. The actuator is controlled by the main control board and by the upper computer software. In order to ensure the stability of the liquid during amplification and droplet preparation, the oil phase used for droplet preparation is thermal curing oil (Shengji Gene Technology Co., Ltd. Xuzhou, China. SXXB No. 20190041), and the oil phase consists of two premixed liquids, A and B, which are mixed well in a 3A:1B ratio before use. The prepared oil phase must be placed on ice and used within 30 min. After the chip loading is completed, the chip is taken out and the valves removed. Finally, the chip is transferred to a flat-plate PCR instrument for dPCR.

### 2.4. PCR Condition

In order to better verify the performance of the KRAS mutation detection system and the performance of the chip designed in this work, a plasmid vector is used for template construction. First, the plasmid template is extracted from the strain containing the wild-type KRAS gene plasmid (provided by Little Turtle Technology Co., Ltd., Shanghai, China), and then the Fast Site-Directed Mutagenesis Kit (Tiangen Biochemical Technology Co., Ltd., Beijing, China) is used to construct mutant strains according to the instructions. The mutation primer system shown in Table S3 is used for PCR amplification, and the amplified plasmid will be mutated according to the designed primer mutation type, and then the mutant plasmid will be introduced into DH5α competent cells. After culturing on the LB (Luria-Bertani) plate medium added with ampicillin (100 ng/mL), pick the single colony that grows for liquid culture, and then carry out plasmid extraction and gene sequencing analysis (Bioengineering Technology Co., Ltd., Shanghai, China) for the cultured mutant strains to identify whether the constructed mutant strain meets the expected requirements according to the sequencing results. The required mutant plasmid template can be obtained by expanding and re-culturing the strains that meet the sequencing requirements and, finally, extracting the plasmid with new bacterial liquid, which lays a solid foundation for the construction and verification of the subsequent detection system. For dPCR amplification, all components of the PCR mixture, including the PCR master mix, primers, probe, and template, were pre-mixed outside of the chip before dPCR analysis. The chip was then placed on a flat apparatus for PCR reaction. To assess the performance of the proposed dPCR chip, KRAS wild-type plasmid DNA was serially diluted, ranging from 2 × 10^1^ to 2 × 10^4^ copies/μL. The dPCR reaction mixture contained a 10× dPCR Buffer 2 (µL), KRAS forward primer (1 µL) (400 nM), KRAS reverse primer (1 µL) (400 nM), KRAS probe (1 µL) (400 nM), serially diluted template (3.5 µL), and RNase-free water (23.2 µL). All DNA samples and reaction mixes were stored at −20 °C prior to use. The PCR primers and probes were synthesized by Sangon Biotech (Shanghai) Co., Ltd.; the primers/probes and reagent preparation are listed in Tables S1, S3 and S4. The thermo-cycling condition included a 10 min heating step at 50 °C to ensure the solidification of the oil phase in the chip and a 10 min activation step at 90 ℃ to initiate Taq DNA polymerase, and then 45 thermal cycles were performed (20 s at 95 °C, 40 s at 58 °C) to amplify the KRAS gene target [26]. All experiments were repeated a total of three times.

### 2.5. Image Acquisition and Analysis

All bright-field images were acquired with an upright fluorescence microscope (Olympus Corporation, Optical Technologies Inc., Tokyo, Japan), and all fluorescence images were acquired and analyzed using a biochip reader system provided by Shengji Gene Technology. Ltd. (Xuzhou, China).

## 3. Results and Discussion

### 3.1. Principle of KRAS Gene Mutation Detection by Multicolor Fluorescence dPCR

A multicolor fluorescence system was used to simultaneously detect different mutation types of KRAS genes, thus simplifying the experimental operation process and reducing the experimental cost. First, the DNA samples and reagents are mixed in a centrifuge tube according to the required ratio of the experiment, and the prepared PCR reaction reagents are divided into the chip through the automated sampling system. The chip is then transferred to a flat-plate PCR instrument (Shengji Gene Technology Co., Ltd., Xuzhou, China, SXZZ No. 20192221303), and PCR amplification is carried out according to the preset procedure. After the amplification, a biochip reader (Shengji Gene Technology Co., Ltd., Xuzhou, China, SXZZ No. 20192221302) is used to detect and analyze the fluorescent signal of the chip. Different from other technical schemes, this work is based on a four-color fluorescence system, which can simultaneously collect four different fluorescence signals for parallel detection of multiple mutations. Three different probes are designed for three mutant types at one site, and each probe is labeled with different fluorescein. The corresponding fluorescein can excite corresponding fluorescence signals under corresponding fluorescence channels. Fluorescence signals are independent of each other and can be independently distinguished after being collected by different filters. According to different fluorescent signals, multiple mutation types can be detected simultaneously on a single chip without mutual interference. In addition, in order to detect samples more accurately, a reference probe is also designed in this scheme. The reference probe can detect all KRAS genes, that is, to interpret the mutation rate of a mutation type, it is necessary to comprehensively consider the signal of the reference probe and the signal of the mutation probe (for example, if the mutation type is positive, both the Cy5 channel and the FAM channel need to have positive signals). With this detection, false positives caused by impurity interference or false negative signals generated by irregularities in the sampling process can be excluded. The detected mutation rate of a specific type of KRAS is calculated as Pm = (mutated copy number)/(reference copy number).

### 3.2. Detection of KRAS Gene Mutation by Multicolor dPCR

An important indicator of mutation detection is that the signal of the mutation probe will not cause non-specific cleavage of the probe in other mutation and wild types, and will not produce signals that interfere with each other. Since the vast majority of clinical mutation types have only one mutation, the six mutation (M) types’ G12S, G12C, G12R, G12D, G12V, G12A templates and wild (W) templates are mixed as simulated mutation samples. All primers and probes are listed in Table S3. In order to be able to distinguish multiple mutation types at once, multiple mutation probes and universal probes were directly mixed. The droplet preparation automatic instrument was then placed inside the chip and transferred to the plate PCR amplifiers for amplification. During the amplification process, the DNA polymerase will cleave the specific hydrolysis probe bound to the target nucleic acid molecule, and the probe hydrolyzed by the enzyme will release free fluorescent groups. At the end of the amplification, the positive chambers will contain a large number of fluorophores. Positive and negative chambers can be easily distinguished by fluorescence microscopy. After the amplification, chip imaging and signal analysis are performed by a fluorescence signal reader.

### 3.3. Performance Analysis of Reagent Segmentation for Microfluidic Chips

In order to verify the liquid dispensing performance of the chip, first, red ink is used instead of reagent to prepare droplets. After the droplet preparation process is completed by using the automatic droplet preparation device, photographing is performed on the optical microscope. The experimental results show that all the chambers were perfectly filled with red ink, and there was no red ink residue in the main channel, indicating that the red ink in all microchannels was replaced by oil. In addition, all the chambers were uniform, which fully proved that the chip designed in this work can be used for droplet preparation and can be used for dPCR.

### 3.4. Primer Evaluation of the Mutation Detection System of KRAS Gene

In order to verify whether the designed KRAS gene mutation detection primer can achieve KRAS template amplification, electrophoresis analysis is performed on the amplified products. The gel electrophoresis bands are shown in Figure S1. Six mutant plasmids and one wild type of plasmid have clear and single electrophoresis bands with a strong brightness, and the length is consistent with the designed primer pair length (100bp), indicating that the primers have good specificity and high efficiency.

### 3.5. Analysis of Mutation Results of KRAS Gene Detected by Multicolor dPCR

**Figure 5:**
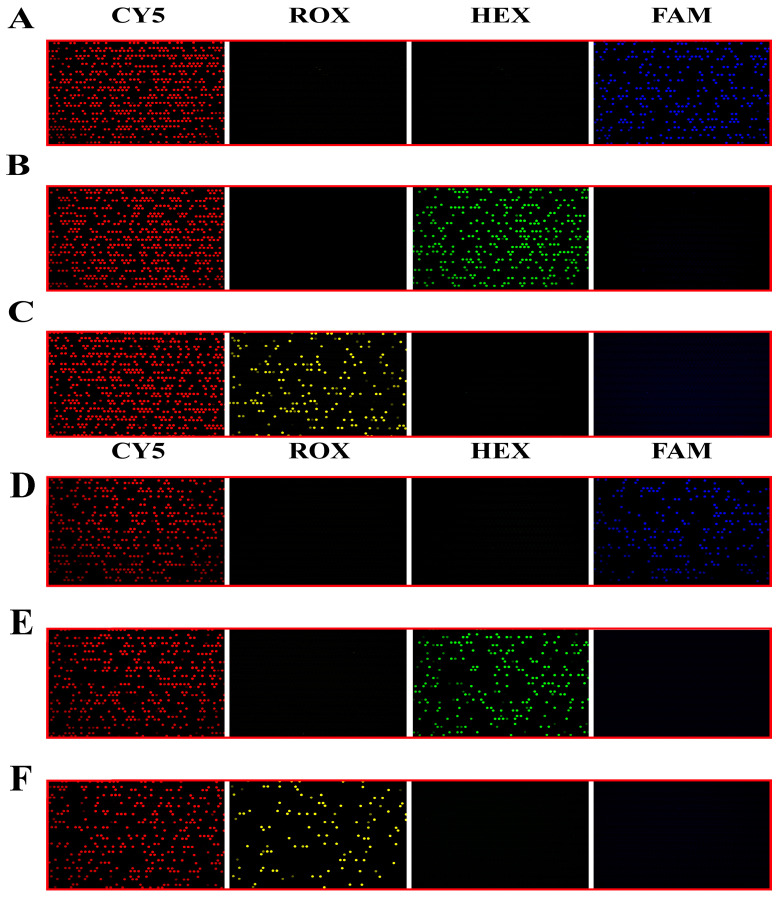
Chip 1 and Chip 2 detection results (the simulated sample concentration is 2 × 10^4^ copies/μL). (**A**) Detection results of G12S wild type of mutant mixed template in four probe systems. (**B**) Detection results of G12C mutant wild mixed template in four probe systems. (**C**) Detection results of G12R mutant wild mixed template in four probe systems. (**D**) Detection results of G12V mutant wild mixed template in four probe systems. (**E**) Detection results of G12D mutant wild mixed template in four probe systems. (**F**) Detection results of G12A mutant wild mixed template in four probe systems.

The KRAS mutant gene is detected by a four-color fluorescence system. According to the design requirements, three mutation types are detected simultaneously for each chip, and each mutation type is labeled by an independent fluorescent probe. In order to verify the detection ability of different mutants, dPCR was performed by mutant and wild mixed templates according to the experimental protocol designed in Table S3. The results are shown in Figure 5. The Cy5 channel shows the reference probe signal, and it can be seen that all chips have more positive chambers, and the channels added with the corresponding mutation templates also have corresponding mutation-positive signals. The mutation channels signals can be matched to the reference channel. In addition, there are obvious differences in the signal strengths of the positive chamber and the negative chamber of all the channels, which is convenient for distinguishing and determining the threshold value. All the chips have no non-specific positive signals, indicating that our reaction system has excellent ability to distinguish mutant subtypes, and it is possible to detect three mutation types on one chip, which is in line with the previous experimental assumption that six mutations needed to be detected by two chips.

### 3.6. Quantitative Analysis of dPCR

**Figure 6:**
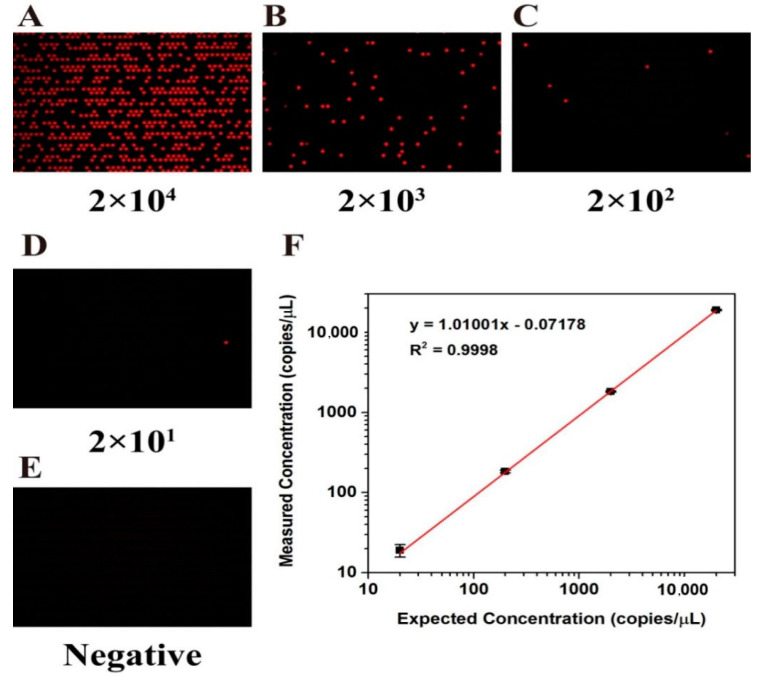
Reactions of gDNA samples at different H1975 cell line concentrations on chip. (**A**–**D**) Fluorescence photographs after serial dilution of DNA template into 2 × 10^4^ to 2 × 10^1^ copies/μL for dPCR amplification. (**E**) Negative control group without DNA template. (**F**) Linear relationship between the number of template copies detected by the dPCR chip and the number of theoretical template copies.

In order to verify the ability of the chip for nucleic acid quantification, the wild-type KRAS plasmid was used for the dPCR experiment. The stock solution of the KRAS wild plasmid template was diluted tenfold into four templates of different orders of magnitude (2 × 10^1^ to 2 × 10^4^ copies/µL) for later use. After the dPCR reaction was completed, according to the fluorescent markers carried by the probes, fluorescence imaging analysis was performed under the Cy5 fluorescence channel by using a signal reader. Fluorescence photographs of the chips after thermal cycling with different concentrations of DNA template are shown in Figure 6A–D. When the concentration of DNA template in PCR is diluted, the number of positive droplets decreases proportionally. There are no positive signal points in the graph of the negative control group (Figure 6E), so it can be inferred that there are almost no pollutants that can cause signal interference in PCR samples.

Quantitative analysis of nucleic acid molecules was carried out according to the Poisson distribution Formula (1), and the mean value of target DNA contained in each microchamber can be calculated according to the actual number of positive microchambers. The test results show that the actual measured nucleic acid concentration and theoretical concentration have good consistency (R^2^ = 0.9998, Figure 6F)

(1) $$C=-\frac{ln(1-\frac{d}{n})}{V_{d}}$$

where C is the concentration of target DNA in the sample, n is the total number of microchambers of the chip, d is the total number of all positive microchambers, and $V_{d}$ is the volume (μL) of each microchamber.

### 3.7. Analysis of KRAS Gene Mutation Detection by dPCR

**Figure 7:**
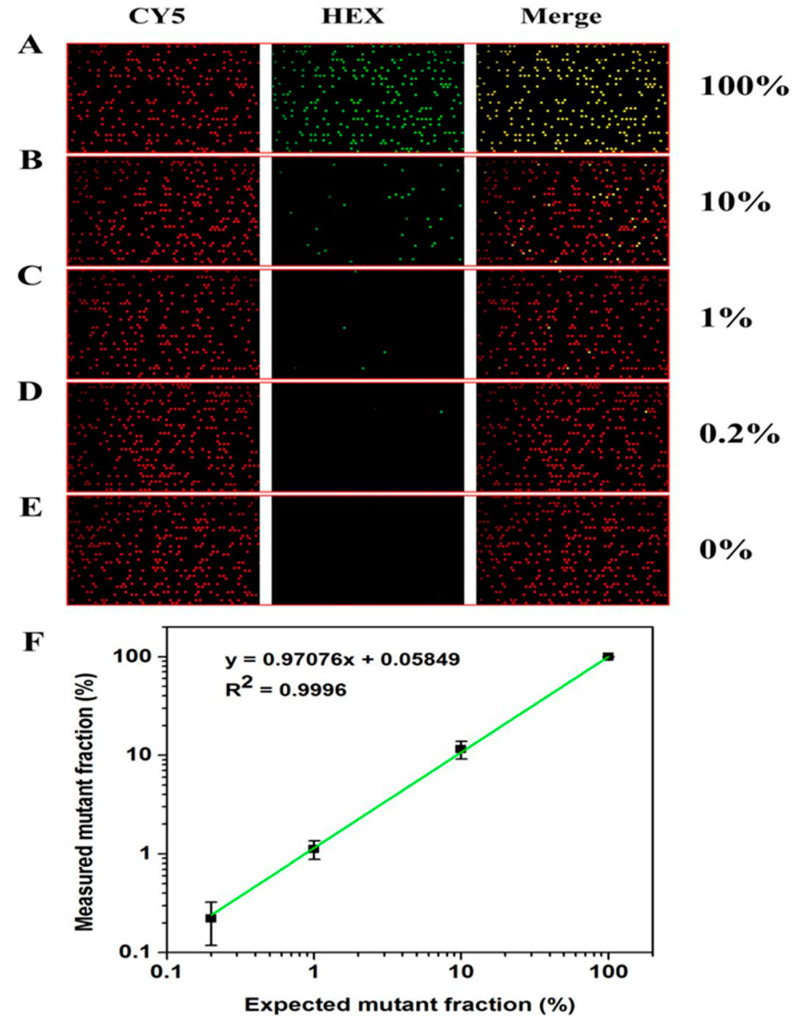
Reaction results of simulated samples with different mutation rates on dPCR chip. (**A**–**E**) Fluorescence photos of G12D simulation samples after amplification under different mutation rates (100%–0). (**F**) Linear relationship between theoretical mutation rate and test mutation rate of simulation samples with mutation rate of 0.2–100%.

Mutation detection is an important application of dPCR, especially single-molecule mutation detection in the background of a highly homologous wild-type DNA sample. In this section, the simulation samples obtained after mixing the G12D mutation template and wild-type template are used to verify the mutation detection performance. As shown in Table S3 and Figure 7, the reference probe and G12D mutation probe are designed in the conservative section and mutation section of the KRAS gene, respectively. The analysis shows that the chambers containing the G12D mutant template are Cy5+ and HEX+, the chambers containing the wild-type template are Cy5+ and HEX−, and the chambers containing no template are Cy5− and HEX−.

The mutation rate is calculated according to Formula (2):

(2) $$P=\frac{C_{HEX}}{C_{Cy5}}\times 100\%$$

where P is the mutation rate of G12D in the simulation sample, $C_{HEX}$ is the concentration of the G12D template in the simulation sample, and $C_{Cy5}$ is the total concentration of the KRAS gene template in the simulation sample.

Figure 7 shows the results of sample detection with different mutation concentrations. It can be seen that the number of positive chambers in G12D decreases with the decrease in the concentration of the mutation template (Figure 7A–D). The chambers containing the mutation template can still be detected with a mutation rate as low as 0.2% (Figure 7D). The G12D positive dot is not detected in the chip without mutation samples (Figure 7E), indicating that the system can well distinguish negative samples from low-copy positive samples. Moreover, there is an excellent linear relationship between the theoretical mutation rate and the actual detected G12D mutation rate (Figure 7F), which once again proves that the chip can accurately and quantitatively detect nucleic acids and is suitable for dPCR applications.

## 4. Conclusions

In this work, we designed a microfluidic chip composed of a microchambers array, which can achieve the microsegmentation of reagents through an automated platform. In addition, the glass “sandwich” structure of the chip ensures that PCR amplification reaction can be stably carried out through the chip, and the good light transmission of the glass is particularly suitable for collecting the fluorescence signal of the chamber after amplification. The flexible valve control technology ensures that the chip can complete the reagent segmentation process through an automated platform. Using the chip designed in this work, a multicolor dPCR technology was developed; the mutation detection system constructed by dPCR can simultaneously detect three KRAS gene mutations on one chip. In addition, the linear quantitative detection proves that the chip can realize absolute quantitative detection of nucleic acid in dPCR, with high precision and sensitivity, and the dynamic detection range can reach 10^4^. This mutation detection result shows that the multicolor dPCR detection technology based on this chip can meet the mutation rate detection precision of at least 0.2%. The detection that originally needs six chips can be completed by this system with only two chips, thereby greatly simplifying the workflow, improving the throughput, and reducing the use of consumables and reagents.

Currently, microfluidic technologies have been applied as a one-stop solution for dPCR to overcome limitations, such as contamination risks associated with manual-pipetting sample transfer, and limited assay precision with more interference from wild genes, which can lead to the occurrence of missed inspection. dPCR not only offers precise absolute quantification of targets without the requirement of calibration curves, but also shows better repeatability at a low target concentration and greater tolerance to inhibitors. The next 5–10 years will be crucial for the growth of the dPCR market demand. A microfluidic chip consisting of a microchamber array has the great ability to make positive changes in the biotech and medical fields.

## Figures and Tables

**Table 1:** Mutation frequency of KRAS gene in cancer.

Original Tissue	KRAS Mutation Rate (%)
Pancreas	90
Colon	30–50
Small intestine	35
Biliary tract	26
Uterine endometrium	17
Lung	19
Skin (melanin)	1
Uterine cervix	8
Urethra	5

## Data Availability

All datasets presented in this study are included in the article/Supplementary Material.

## Supplementary Materials

The following supporting information can be downloaded at https://www.mdpi.com/article/10.3390/bios13080778/s1: Table S1. Reagents and instruments required for the construction of KRAS mutation gene detection system. Table S2. Main reagent materials and instruments in the chip preparation process. Table S3. Sequences of primers and probes used in this experiment. Table S4. Preparation of dPCR reaction for KRAS gene mutation detection and preparation of dPCR reaction for linear quantitative detection and analysis. Figure S1. Gel electrophoresis diagram after PCR amplification of the constructed mutant plasmid.
