# Supplementary material for: A Chamber-Based Digital PCR Based on a Microfluidic Chip for the Absolute Quantification and Analysis of KRAS Mutation
Source: Biosensors (Basel). 2023 Aug 1;13(8):778. doi: 10.3390/bios13080778 (PMC10452697; doi:10.3390/bios13080778)

biosensors

Supplementary Materials

A Chamber-Based Digital PCR Based on a Microfluidic Chip for the Absolute Quantification and Analysis of KRAS Mutation

Jie Ren [1,†], Gangwei Xu [2,3,†], Hongna Liu [1,†], Nongyue He [1], Zhehao Zhao [3], Meiling Wang [1], Peipei Gu [1], Zhu Chen [1], Yan Deng [1], Dongping Wu [2,3,*] and Song Li [1,4,*]

Table S1 Reagents and instruments required for the construction of KRAS mutation gene detection system.

Reagent/instrument name	Manufacturer/source
Wild-type KRAS gene plasmid strain	Turtle Tech Ltd., Shanghai, China
Plasmid Midi Preparation Kit	AxyPrep, Corning Life Sciences Co., Ltd
Fast Site-Directed Mutagenesis Kit	Tiangen Biochemical Technology (Beijing) Co., Ltd.
Competent DH5α strain	Shanghai Shenggong Technology Co., Ltd
Peptone	Sigma Aldrich, USA
Yeast extract	Sigma Aldrich, USA
NaCl	Sigma Aldrich, USA
Desktop small centrifuge	DLAB Scientific Co., Ltd.
High speed centrifuge	Thermo Fisher Scientific, USA
Thermostatic water bath	Shanghai Lingfeng Chemical Reagent Co., Ltd.
Pipette	Eppendorf, USA
Thermostatic bacterial incubator	Shanghai Heheng Instrument Equipment Co., Ltd.
LB medium	Turtle Tech Ltd., Shanghai, China

Table S2. Main reagent materials and instruments in the chip preparation process.

Reagent/Instrument name	Manufacturer
Concentrated sulfuric acid	Sinopharm Chemical Reagent Co., Ltd. (Shanghai)
Anhydrous ethanol	Sinopharm Chemical Reagent Co., Ltd. (Shanghai)
Acetone	Sinopharm Chemical Reagent Co., Ltd. (Shanghai)
SU8-3050 photoresist	Micro Chem, USA
PGEMA developer solution	Micro Chem, USA
Polydimethylsiloxane (PDMS)	Sylgard 184, Dow Corning, USA
Triton X-100 (TX-100)	Sigma Aldrich, USA
Spin coater	Laurell Technologies, USA
Hot plate	Suzhou CChip Scientific Instrument Co., Ltd.
Ultra violet lithography	NXQ, USA
Plasma cleaner	Suzhou CChip Scientific Instrument Co., Ltd.
Upright fluorescence microscope	Olympus, Japan

Table S3. Sequences of primers and probes used in this experiment.

Mutation type		Primer sequence (5'-3')
G12S	Forward primer	GTAGTTGGAGCTAGTGGCGTAGGCAAG
	Reverse primer	CTTGCCTACGCCACTAGCTCCAACTAC
G12C	Forward primer	GTAGTTGGAGCTTGTGGCGTAGGCAAG
	Reverse primer	CTTGCCTACGCCACAAGCTCCAACTAC
G12R	Forward primer	GTAGTTGGAGCTCGTGGCGTAGGCAAG
	Reverse primer	CTTGCCTACGCCACGAGCTCCAACTAC
G12D	Forward primer	GTGGTAGTTGGAGCTGATGGCGTAGG
	Reverse primer	CCTACGCCATCAGCTCCAACTACCAC
G12V	Forward primer	GTGGTAGTTGGAGCTGTTGGCGTAGG
	Reverse primer	CCTACGCCAACAGCTCCAACTACCAC
G12A	Forward primer	GTGGTAGTTGGAGCTGCTGGCGTAGG
	Reverse primer	CCTACGCCAGCAGCTCCAACTACCAC

Mutation type	Reporter fluorochrome	Probe sequence (5'-3')
Reference probe	Cy5	AGAGTGCCTTGACGATACAGCTAA
G12S probe	FAM	AGCTAGTGGCGTAG
G12C probe	HEX	AGCTTGTGGCGTAG
G12R probe	ROX	TGGAGCTCGTGGCGTAG
G12D probe	HEX	AGCTGATGGCGTA
G12V probe	FAM	AGCTGTTGGCGTAG
G12A probe	ROX	AGCTGCTGGCGTAGGC

Table S4. Preparation of dPCR reaction for KRAS gene mutation detection.

Reagent component	Volume (35 μL)
10×dPCR Buffer	3.5 μL
Enzyme	1 μL
Upstream primer	1.4 μL (400 nM)
Downstream primer	1.4 μL (400 nM)
Reference probe	1 μL (400 nM)
G12S probe	1 μL (400 nM)
G12C probe	1 μL (400 nM)
G12R probe	1 μL (400 nM)
Simulation sample (1 M template + W template)	3.5 μL
Nuclease-free water	20.2 μL

Preparation of dPCR reaction for linear quantitative detection and analysis.

Reagent component	Volume (35 μL)
10×dPCR Buffer	3.5 μL
Enzyme	1 μL
Forward primer	1.4 μL (400 nM)
Reverse primer	1.4 μL (400 nM)
Reference probe	1 μL (400 nM)
Wild-type template	3.5 μL
Nuclease-free water	23.2 μL

Figure S1. Gel electrophoresis diagram after PCR amplification of the costructed mutant plasmid-G12S, G12C, G12R, G12D, G12V, G12A and the wild plasmid-WT.

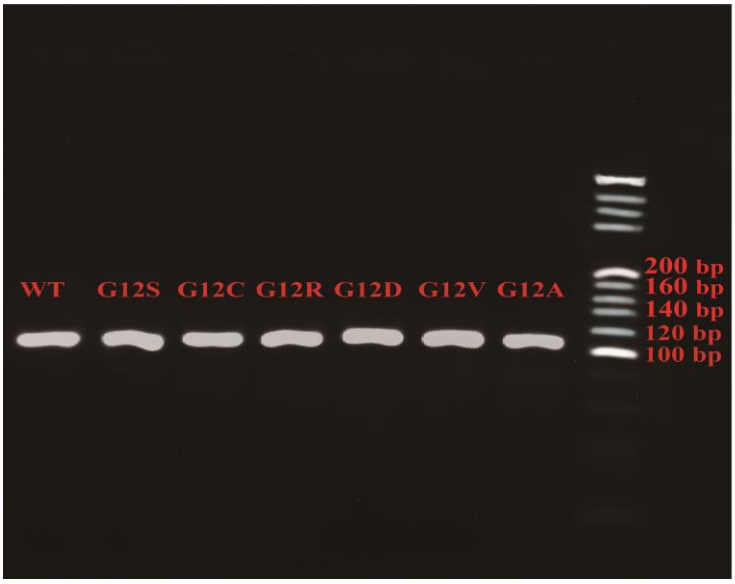

Supplement: Supplementary file 1 [file biosensors-13-00778-s001.zip › biosensors-2489644-supplementary.pdf]
